# Design and evaluation of a sustainable entropy-weighted and VIKOR-based method for offshore oil collecting

**DOI:** 10.1016/j.heliyon.2023.e21256

**Published:** 2023-10-20

**Authors:** Suwen Luo, Pengrui Yang

**Affiliations:** School of Art, Hubei University, Wuhan City, China

**Keywords:** Sustainable development, Offshore oil collection design, Entropy weight method, VIKOR, Design model

## Abstract

With the expansion of marine oil exploitation, offshore oil leakage has become an urgent problem that cannot be ignored in marine ecological protection. To address the problem, this paper proposes a comprehensive design and evaluation approach aiming at the design of offshore oil collection. This approach combines the entropy weight and VIKOR method with three principles of sustainable design. Specifically, to establish the evaluation model, this paper transforms these principles of sustainability into three aspects of practicality, economy, and environmental protection, thus constructing a multi-level evaluation index system. Subsequently, entropy weights are then used to decline the one's own element in the decision maker's weights. Besides this, the VIKOR method is applied to acquire the optimal ranking of design alternatives for offshore oil collection to choose the optimal product design strategy. According to the obtained experimental results, the proposed design method combining the entropy weight and the VIKOR method is feasible and reasonable. To be exact, it improves the comprehensive index of the product in the design concept stage by over 20%, and it effectively optimizes the resource allocation of the design process.

## 1 Introduction

With soaring population, resource shortage and environmental deterioration, the world has entered a new period of sustainable exploitation and utilization of marine resources [1]. The increase in the amount of oil accidentally released into the marine environment, due to industrial emissions from coastal areas around the world and an increase in offshore transport accidents, means that oil leakage treatment schemes and response strategies need to change [2]. In the last decades, countries have started to explore relevant solutions in response to the threat of oil pollution at the sea surface. In this regard, sustainable design has provided new methods that strike a balance between environmental, economic, and social requirements. As a strategic approach enabling sustainable transformation, the sustainable design has been actively leading the design of green products, services, buildings, environments, and social systems [3].

For the past few years, numerous researchers have applied different methods to the development of sustainable products. For example, Dawodi et al. [4] selected the optimal concept through the technique for order preference by similarity to an ideal solution (TOPSIS), the distance between ideal and non-ideal solutions. Differently, Goswami and Tiwari [5] designed a framework using Bayesian network method to select a relatively optimal design concept with minimal risk. Additionally, Zhu et al. [6] and Tiwari et al. [7] proposed an integrated approach by using VlseKriteriumska Optimizacija I Kompromino Resenje (VIKOR) method, in order to perform multi-criteria decision analysis to select the premier design concept. Besides this, Shidpour et al. [8] pointed out a multi-criteria design evaluation method based on rough set theory for evaluating quantitative criteria of products (e.g., cost) and fuzzy set theory for evaluating qualitative criteria (e.g., aesthetics). Furthermore, Rondini et al. [9] proposed an approach based on two-step materiality-performance analysis, which focuses on product-service systems.

Although the aforementioned methods using sustainable design concepts have improved the green nature of products to some extent, they still have some room for improvement, including but not limited to 1) the lack of a method that can effectively sort out and quantitatively analyze various uncertainties affecting the design objectives when performing multi-criteria decision analysis; 2) due to the subjectivity of design understanding, the planning process needs to pay attention to the combination of objective and subjective, thereby reducing the impact of human and other objective factors. Therefore, we propose a novel attempt to combine the entropy right and VIKOR approaches for the sustainable design. Specifically, the entropy weighting method reflects the true value of entropy information in the sample and does not add subjectivity, hence the index weight obtained by it can be more objective. VIKOR, on the other hand, is able to rank the evaluation schemes according to their qualities and get the ideal plan that is closest to the design goal, thus maximizing the utility of the group [10].

The marine environment is an important pathway for the well-being of human society and for economic innovation and sustainable development, and understanding the feasibility of how to adopt and utilize technological approaches as a solution to the practical problems of oil spills is critical to the national and global environment, and provide reference value for relevant marine pollution treatment methods.

By summarizing the existing relevant research methods, we found that in the existing design research, the issue of the attribute framework of the specific design product is not considered, resulting in a lack of detailed direction guidance based on the classification of needs in the design process. To address this gap, this paper explores the methodology and design practice of offshore oil recovery product solutions on the basis of existing research, guided by the concept of sustainable design and the integrated application of entropy right and VIKOR methods. The conclusion shows that the advantage of this research is that the method presented in this article effectively improves the functional indexes of the product at the design stage while ensuring the accuracy of the data conclusions, saves the time of the preliminary data collection and processing, effectively optimizes the design steps, and shortens the design cycle.

We get the ideal solution closest to the design goal by this method and get a sea surface oil collection product. The product is based on intelligent algorithms and AI remote control technology, and is capable of realizing outdoor autonomous oil collection through physical oil-water separation in a specific operating environment. The product was compared with different algorithms to obtain the optimal consistency conclusion, thus confirming the feasibility and robustness of the current research methodology. The conclusion of this validation can provide new paths and ideas for the application of related products, so as to maximize the reference value for the development of related oceans.

The main contributions of this research are as below. 1. We enhance the VIKOR methodology, combined with the entropy weight method, which, in the framework of the sustainable concept, makes up for the problem of the lack of the attribute subordination framework of the product in the existing research methods. 2. We apply the improved methodology to the design process of sea surface oil collection, and the results show that the method effectively saves the time of pre-processing data, optimizes the design steps, and improves the design efficiency. 3. The sea surface oil collection product we designed verifies the feasibility of the method proposed in this paper, and designs a product that can solve the problem of sea surface oil pollution and provide reference value for related design practice.

The rest of the paper is structured as follows. Section 2 explains the methods of sustainability, entropy, and VIKOR. Section 3 explicates the components and operation steps of the proposed model. Section 4 describes the evolutionary process of the input data of the offshore oil collection design model and the data comparison of different methods. Finally, Section 5 provides a summary, managerial and theoretical implications, limitations and research topics.

## 2 Theory and methods

**Figure 1:**
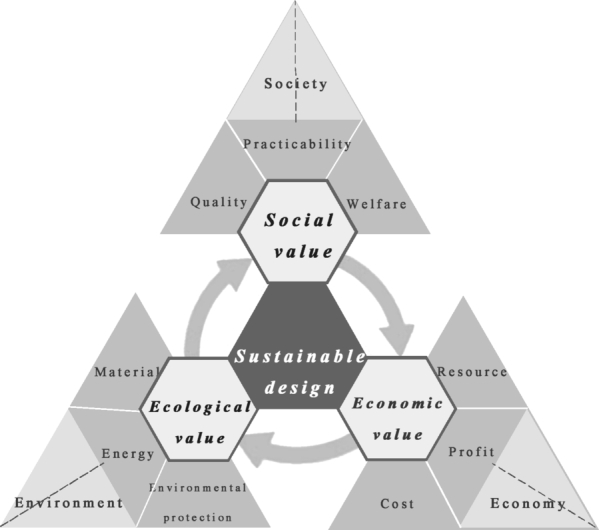
Sustainable framework system.

Sustainable design is a process of profound reflection and continuous change in the practice of the design community on the relationship between human development and environmental issues [11]. It is often defined as a way of development that meets current needs without compromising the ability of future generations to meet their own needs [12]. Sustainable design involves three interrelated pillars, i.e., social, economic, and environmental [13]. These pillars are usually expressed in terms of meeting the need for economic growth while enhancing ecological benefits and social well-being. Therefore, the three principles of sustainability have to be followed in the design process, and this translates into the three values shown in Fig. 1. We expect to find an optimal way to solve the problem through a new technological approach to sustainability that will lead to the perfection of the product in the design phase.

At present, the comprehensive evaluation method combining multi-attribute and multi-factor has become the main research direction of evaluation methods, among which entropy weight method is a typical representative [14]. In the application of entropy weighting method, where the weight of each indicator is obtained based on the differentiated size of the data and through the formula of entropy weight [15]. In applying this method, experts will evaluate the practical conditions of the project and obtain a scoring matrix. Afterwards, experts carried out data standardization management on the obtained scoring matrix, solved the entropy of each index, and calculated the weight of each index [16]. However, there are a number of influencing factors that arise during the product design process, such as the multi-criteria decision-making problem (MCDM). VIKOR is the optimal trade-off solution for multi-criteria decision making proposed by Opricovic et al. in 1998 [17], [18]. However, there are drawbacks to using the VIKOR method alone and it needs to be combined with other methods to solve different problems.

The commonly used evaluation and optimization methods of design schemes include the analytic hierarchy process, fuzzy evaluation, approximate ideal solution ranking and entropy weight method. In recent years, the entropy weight method and VIKOR method have been increasingly applied in the fields of industrial design and economic management [19]. In particular, VIKOR method has a great advantage in obtaining the incongruity between ideal scheme and data processing, and it has been widely applied in multi-criteria decision problems [20]. Therefore, in this paper, the evolution of sustainable design theory will be studied from the perspectives of process, system, multi-scale and multi-dimension, and the entropy weight and VIKOR approaches will be merged into construct the evaluation index of the model. Specifically, on the one hand, the method proposed in this paper will use the entropy weight method to obtain the objective weights of each indicator. On the other hand, the VIKOR methodology is used to rank the best alternatives to maximization collective benefits and minimization individual regret trade-offs, thereby filtering out the optimal product design solution model.

**Figure 2:**
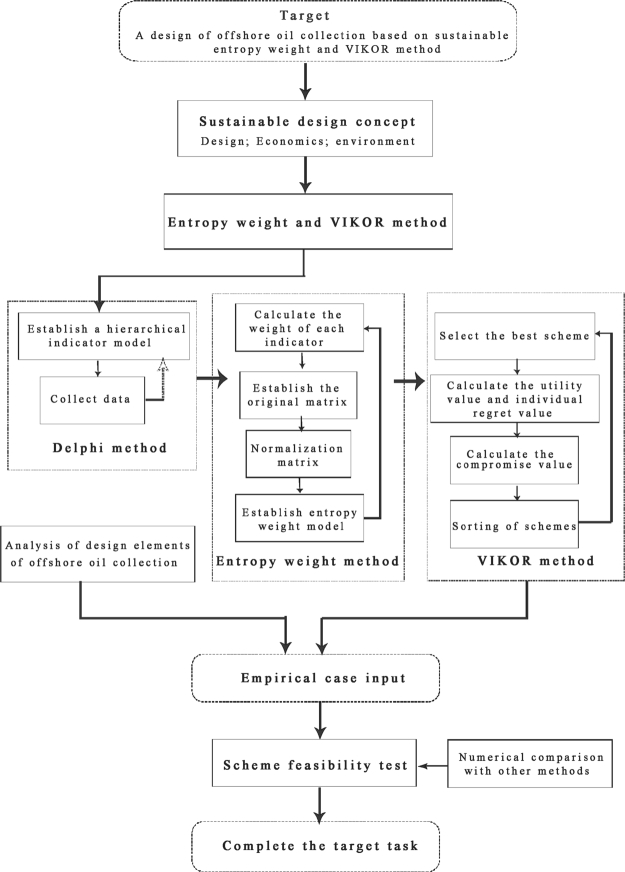
The flowchart of the proposed method.

In the design process of traditional offshore oil collector, there are some problems in the preliminary design, i.e., fuzziness and complexity. This is because the development process of offshore oil collection design is affected by various objective factors, including environment, designers and users. In this context, it is difficult to determine empirically the key factors affecting design options, but the method we mention in the paper can alleviate this problem to a certain extent. They can eliminate the subjective elements of decision makers in the weighting of programs, obtain the objective weighting of indicators, and avoid the subjectivity and randomness of weight decision. Fig. 2 depicts the flowchart of the proposed method, that can be applied to obtain the optimal ranking of candidates that maximizes group benefits and minimizes individual regret compromises. In addition, this method can reduce the influence of human factors and environmental factors on decision makers in the design process. In view of this, with sustainable development as the starting point, we will present a method combining entropy scaling and VIKOR for evaluating and optimizing design solutions.

## 3 Mathematical model

### 3.1 Evaluation index model construction

Optimization and evaluation of product design is a complex multivariate decision problem. Additionally, the selection of assessment indicators is characterized by completeness and non-overlapping. Current products related to offshore oil collection can be divided into two main categories. One is the emergency disposal equipment attached to large ships, and the other is the large marine garbage collector. The main function of the latter is to collect floating rubbish on the sea surface and realize the cleaning of offshore oil. Through research, we found that offshore oil collection equipment needs to meet two requirements. The first one is that these devices need to conform the basic function of clean oil collection on the surface, and add additional water quality monitoring and intelligent detection functions to increase the value of the product. The second point is that in the later maintenance of the product, the operation convenience and the cost of these equipments need to be fully considered.

**Figure 3:**
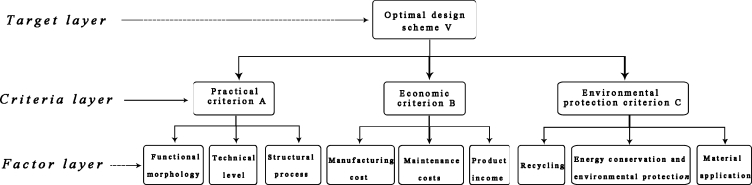
Index evaluation system of offshore oil collection design.

To meet the above requirements, we plan to analyze and discuss the collected data and evaluation index factors through several rounds of Delphi method, and finally get an evaluation model of offshore oil collection product design. Firstly, by analyzing the obtained demand factors, an optimal design evaluation structure model is designed. The specific structure of this model is shown in Fig. 3, which is mainly divided into the target layer, criterion layer and factor layer. Then, an evaluation system of offshore oil collection platform, as shown in Table 1, is established from three perspectives of practicality, economy, and environmental protection.

**Table 1:** Evaluation index system of offshore oil collection design.

	A1 Functional form	Benefit type
A Practicality	A2 Technical level	Benefit type
	A3 Structural technology	Benefit type

	B1 Manufacturing cost	Benefit type
B Economy	B2 Maintenance cost	Benefit type
	B3 Product benefit	Benefit type

	C1 Recycling efficiency	Benefit type
C Environmental protection	C2 Energy saving and environmental protection	Benefit type
	C3 Material use	Benefit type

### 3.2 Calculation of indicator weights

According to the design index evaluation system shown in Fig. 3, we have invited some product design experts to give relative scores with numbers 1–9 and reciprocal scales, so as to determine the relative importance of each index. The main steps are as follows.

1) Establish the original matrix. By comparing the importance of each element between the two levels, this paper establishes a judgment matrix as shown in Table 1. According to VIKOR's theory, the structure of matrix *A* is *m* rows and *n* columns. This means that have *m* programs in the scoring system and *n* scoring indicators in each program as shown in equations (1). Evaluated values for each solution metric *m* are represented by *aij*.

(1) $$A=\left(\begin{array}{cccc} a_{11}\; & a_{12}\; & \ldots \; & a_{1n}\\ a_{21}\; & a_{22}\; & \ldots \; & a_{2n}\\ \vdots \; & \vdots \; & \; & \vdots \\ a_{m1}\; & a_{m2}\; & \cdots \; & a_{mn} \end{array}\right)$$

2) Normalization matrix. In this step, normalization of matrices using vector normalization methods. Matrix *X* can be obtained by normalizing cost type and benefit type according to equations (2) and (3).

(2) $$xij=\frac{aij}{\sqrt{\sum_{i=1}^{m}a_{ij}^{2}}}$$

where *J* belongs to a benefit type.

(3) $$xij=\frac{\frac{1}{aij}}{\sqrt{\sum_{i=1}^{m}{\left(\frac{1}{aij}\right)}^{2}}}$$

where *J* belongs to a cost type.

(4) $$X=\left(\begin{array}{cccc} x_{11}\; & x_{12}\; & \ldots \; & x_{1n}\\ x_{12}\; & x_{22}\; & \ldots \; & x_{2n}\\ \vdots \; & \vdots \; & \; & \vdots \\ x_{m1}\; & x_{m2}\; & \cdots \; & x_{mn} \end{array}\right)$$

Where *aij* is the assessed value of each index, m=3, and *xij* is the assessed value of each index after normalization. 3) Establish an entropy weight model. After we define the m⁎n evaluation matrix through equations (4), we need to calculate the weights of the *jth* decision indicator attribute values for the *ith* program according to equations (5).

(5) $$pij=\frac{xij}{\sum_{i=1}^{m}xij}$$

where *pij* is the attribute value for decision metrics.

4) Then the entropy of the *jth* decision index is calculated according to equations (6).

(6) $$eij=-k\sum_{i=1}^{m}pij\ln pij$$

where *k* is a constant and $k=\frac{1}{\ln m}$, *eij* is the calculated entropy value of each index.

5) Then calculated each weight value according to equations (7).

(7) $$wj=\frac{1-ej}{\sum_{j=1}^{n}\left(1-ej\right)}$$

Where *wj* is the weight value of each index, and *ej* is the entropy value of the *jth* decision index.

### 3.3 The optimal scheme selection of the model

Utility value *Si* of alternative scheme and regret value *Ri* of individual need to be calculated.

(8) $$Si=\sum_{j=1}^{n}\frac{wj\left(xj^{+}-xij\right)}{xj^{+}-xj^{-}}$$

(9) $$Ri=\underset{j}{\max}\;\left\{\frac{wj\left(xj^{+}-xij\right)}{xj^{+}-xj^{-}}\right\}$$

In Eq. (8) and Eq. (9), $x_{j}^{+}$ is positive ideal solution, $x_{j}^{+}=\underset{i}{\max}\;\left\{xij\right\}$, $x_{j}^{+}$ is negative ideal solution, $x_{j}^{+}=\underset{j}{\mathrm{mix}}\;\left\{xij\right\}$.

Then, the compromise value of alternative schemes should be determined.

(10) $$Qi=\varepsilon \frac{Si-S^{-}}{S^{+}-S^{-}}+\left(1-\varepsilon \right)\frac{Ri-R^{-}}{R^{+}-R^{-}}$$

In Eq. (10), $S^{+}=\underset{i}{\max}\;\left\{Si\right\}$, $S^{-}=\underset{i}{\min}\;\left\{Si\right\}$, $R^{+}=\underset{i}{\max}\;\left\{Ri\right\}$, $R^{-}=\underset{i}{\min}\;\left\{Ri\right\}$.

In the formula, ε,ε∈[0,1], when ε>0.5, when ε<0.5.

Subsequently, the preferred scheme needs to be determined. There are two steps to determining the best solution. 1) The alternative programs are listed in descending order according to the value of SiRiQi; 2) In order of *Q* from smallest to largest, the first program *a*1 meets both of the following requirements. The requirement 1 is $Q\left(a2\right)-Q\left(a1\right)\geq \frac{1}{\left(m-1\right)}$, where *m* represents the number of alternatives. *a*2 is the second scheme arranged according to *Qi* value, and m m represents the number of options. The requirement 2 is the Stability of acceptable decision. Alternative *a*1 must be ranked the best among *Si* or *Ri* and stabilization in decision-making. If condition 1 and condition 2 are met at the same time, then *a*1 is the best program. If only requirement 1 is fulfilled, there exists a set of trade-off solutions {a1,a2}. If only requirement 2 is fulfilled, then there exists a set of trade-off solutions {a1,a2,⋅⋅⋅,am}. The maximization value of m is depend on the relation $Q\left(am\right)-Q\left(a1\right)<\frac{1}{\left(m-1\right)}$, which determines the set of programs for the trade-off solution.

## 4 Scheme and application

### 4.1 Objective case design factors

(1) Function and principle. Table 2 illustrates that existing oil collection methods, the main methods to deal with offshore oil leakage at present include physical treatment, chemical treatment and biological treatment, among which physical treatment is the most effective measure to deal with the problem. According to Table 2, we have listed safe and pollution-free physical oil collection methods and have developed detailed solutions.

**Table 2:** Existing oil collection methods.

Method	Specific measures			Advantages	Disadvantages
Physical Treatment	Containment Boom	Mechanical method	Adsorption method	Simple and effective, Mature technology, Pollution-free	Upfront cost input is required
Chemical treatment	Chemical reagent method	Combustion method		Low cost, Low processing costs, No complexity	It is toxic to the Marine environment and wastes energy
Biological treatment	Bioremediation technology	Bioenhancement technique		The ability of high intensity degradation	Limited by environmental factors

**Figure 4:**
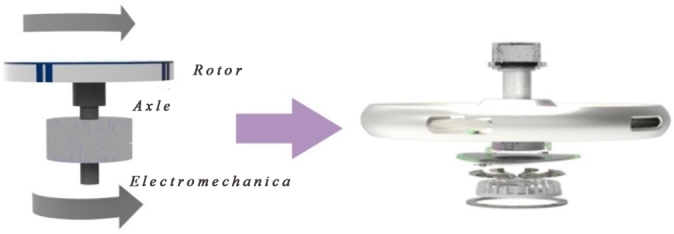
Centrifuge structure diagram.

We conclude that the physical treatment and mechanical devices are the most effective in cleaning up oil spills on the surface and shore. Therefore, the functional principle of the design of offshore oil collection in this paper will be the centrifuge shown in Fig. 4, which is a physical way of separating oil from water. Specifically, it works by taking advantage of the density difference between oil and water and using pumps to extract oil from the oil-water interface to physically collect offshore oil.

(2) Structure and modeling. When designing a structural system, we should identify the product environment in which the structure will be used. The environmental conditions of the objects studied in this paper include mechanical conditions (vibration, acceleration, impact), physical conditions (seawater pressure, temperature), meteorological conditions (wind and rain, lightning, snow and ice) and atmospheric conditions (ultraviolet, fog, wind). In addition, energy consumption is an important factor to evaluate the rationality of product modeling. We can use aerodynamic streamlining to reduce unnecessary energy consumption during operation and maximize work benefits. Therefore, we set the product as a streamlined integrated structure and maximize its structural space to meet the usage requirements. The advantage of this approach is that the product can effectively use the external form to adapt to the use of the environment while combining the shape and function of the product.

(3) Material. The degree of material quality of the designed product in this paper is judged according to the ratio of structural performance to structural weight. If the weight of the structure itself is light, the transport and carrying efficiency is high. Therefore, when the structural weight of the object studied in this paper is relatively light, it is more conducive to its efficient and low-energy driving in the ocean. The main material of this paper is aluminum alloy, which has the characteristics of hard, beautiful, light and durable. In addition, the material's smooth surface helps extend the product lifetime, in line with sustainable design principles.

### 4.2 Input data for the case study

**Figure 5:**
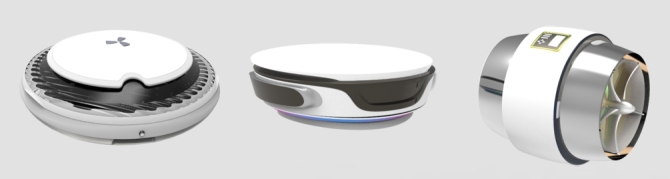
Three designs for offshore oil collection.

Table 3 indicates that the data of the scheme initial evaluation matrix, we established an evaluation index system for offshore oil collection in line with sustainable development, in which industry experts were invited to assign values to evaluation indexes using a 9-level scale and analyzed the scores of each index in SPSS software for reliability and validity. According to the above evaluation indexes, we intuitively understand the design factors of offshore oil collection platform and its importance degree distribution. It can be seen from the data that the design of offshore oil collection platform pays the most attention to the functional criteria and environmental applicability. To match the weight value of the above design and the environmental demand for offshore oil collection design, we start from the modeling factors, through the mechanical structure model to carry out further functional zoning of the product design inside and outside, so as to improve the ease of use of stakeholders. In the following design process, we focus on functional and environmental criteria. In this paper, three design options for offshore oil collection are selected, as shown in Fig. 5.

**Table 3:** Scheme initial evaluation matrix.

Evaluation level	Corresponding evaluation index	Scheme 1	Scheme 2	Scheme 3	Index form
	A1 Functional form	8.2	7.9	7.9	Beneficial type
A Practicality	A2 Technical level	8.0	7.8	8.0	Beneficial type
	A3 Structural technology	8.0	7.7	7.9	Beneficial type

	B1 Manufacturing cost	8.2	7.5	7.6	Beneficial type
B Economy	B2 Maintenance cost	8.0	7.8	8.0	Beneficial type
	B3 Product income	7.8	7.6	7.9	Beneficial type

	C1 Recycling	8.2	7.8	7.7	Beneficial type
C Environmental protection	C2 Energy saving and environmental protection	8.2	7.8	7.9	Beneficial type
	C3 Material application	8.2	7.6	7.8	Beneficial type

The steps of design evaluation, analysis and optimization of these schemes by using the entropy weighting and VIKOR methods are as follows.

(1) Establish the initial evaluation matrix. According to the evaluation index system of offshore oil collection design shown in Table 1, this paper uses a 9-level scale (1–9) and industry experts were invited to assign values to each of the assessment indicators for the three programs. Respectively, then calculates the average values and obtains the initial scheme evaluation matrix *A* as shown in Table 2.

(2) Normalize the evaluation matrix. Based on the calculation methodology provided by VIKOR strategic decision, we have calculated that assessment of all appraisal indicators in the system are in the form of benefit types, so the matrix *X* was obtained after normalization processing according to equation (2) and the standardized assessment matrix data in Table 4.

**Table 4:** Standardized evaluation matrix.

Evaluation index	Scheme 1	Scheme 2	Scheme 3
A1	0.591	0.570	0.570
A2	0.582	0.567	0.582
A3	0.587	0.565	0.579
B1	0.604	0.522	0.574
B2	0.582	0.567	0.582
B3	0.579	0.564	0.587
C1	0.599	0.569	0.562
C2	0.594	0.565	0.572
C3	0.601	0.557	0.572

(3) Calculate the weight *wj* of each index. In this step, the weight value *wj* for each indicator is calculated using Formula (7) as shown in Table 5.

**Table 5:** Weight value of each indicator.

Index	*Wj*	Index	*Wj*
A1	0.0671	B3	0.0557
A2	0.0305	C1	0.1603
A3	0.0543	C2	0.0977
B1	0.2882	C3	0.2158
B2	0.0305	–	–

(4) Determine the best scheme. According to equations (8) and (9), SiRi and *Qi* are calculated for each of the three scenarios and are listed in order from small to large. The numerical values for the program assessment are presented in Table 6.

**Table 6:** Scheme evaluation calculation value.

Scheme code	*Si*	*Ri*	*Qi*
1	0.018	0.019	0.000
2	0.967	0.288	1.000
3	0.627	0.165	0.592

Solutions obtained according to VIKOR's methodology for evaluating the best solutions are ranked in order of median value *Qi* from small to large. Since $Q\left(a2\right)-Q\left(a1\right)\geq \frac{1}{\left(m-1\right)}$ (m=3), condition 1 is satisfied. In addition, the value of a1 is the optimal ordering among the values of *Si* and *Ri* and the cluster and individual regret values are ordered in the same way as the median, thus it fulfills requirement 2. Therefore, the ranking result can be obtained as scheme 1 > Scheme 3 > Scheme 2, i.e., scheme 1 is the best scheme.

Three alternative schemes of offshore oil collection products were tested by the calculation results, and scheme 1 was selected as the optimal model. Subsequently, in order to further match the requirements of the criterion layer, we put forward the following improvement suggestions according to the Table 7 displayed Prioritization of impact factors for the calculation results of Scheme 1 combined with the multi-criterion compromise solution sorting method (VIKOR).

**Table 7:** Value prioritization.

Name	Population effect value (S)	Individual regret value (R)	Profit ratio value (Q)	Sort
A1 Functional form	0.109	0.109	0.000	1
A2 Technical level	0.242	0.148	0.188	3
A3 Structural technology	0.445	0.186	0.435	5
B1 Manufacturing cost	0.592	0.372	0.918	9
B2 Maintenance cost	0.242	0.148	0.188	3
B3 Product income	0.687	0.297	0.857	8
C1 recycling	0.422	0.329	0.688	7
C2 Energy saving and environmental protection	0.203	0.109	0.081	2
C3 Material application	0.499	0.279	0.660	6

1) Increase the environmental applicability of products. We will choose an all-in-one, streamlined shape that is suitable for driving in seawater.

2) Increase the lighting function of the product. We will choose halogen lamps which can increase the visibility of our products. In weather conditions with low visibility, such as rain, snow and fog, halogen lamps have the advantages of better penetration, better lighting effect, higher safety and lower cost.

3) Add a camera and video instrument to the product. When working 24 hours, the product can complete video recording and environmental recording, and upload the video and device information to the cloud for easy data query. In the event of an emergency sea situation, users can reduce their losses by reviewing the data stored in the cloud.

4) Add thermosetting FRP and ABS plastics to the product. On the one hand, thermosetting glass not only has high specific strength, good electrical insulation, stability, but also can be made into any curved surface shape and thickness of different complex forms. On the other hand, ABS plastic has good corrosion resistance, it can maintain good material particularity at higher temperatures, and has the advantages of high surface hardness, high mechanical strength and low cost.

5) Add remote control mode for users. We set two usage modes for the product so that users can better operate and manage the working sea area. The first mode is the daily mode, that is, the low-energy state, which can monitor the environmental state of the working area in real time and collect the water quality test data of the monitoring area. The second mode is the emergency mode, which occurs when there is an oil spill. In this mode, the user can detect oil spills on the sea surface through the camera, and the product immediately starts the emergency mode for emergencies and quickly completes the oil collection in the designated area.

### 4.3 Optimization model display

Fig. 3 shows the final effect obtained after we make the above optimization suggestions for the screened scheme 1. On the one hand, the function of this product design needs to meet the daily work of the sea water sample detection. On the other hand, it can collect and recover oil from the spilled sea area in case of oil spill accident. The product automates the detection and collection of spilled oil while reducing the risk of oil spreading with ocean currents. From the perspective of functional innovation, it adds alarm system and real-time monitoring function, providing more possibilities for offshore oil collection platform equipment.

**Figure 6:**
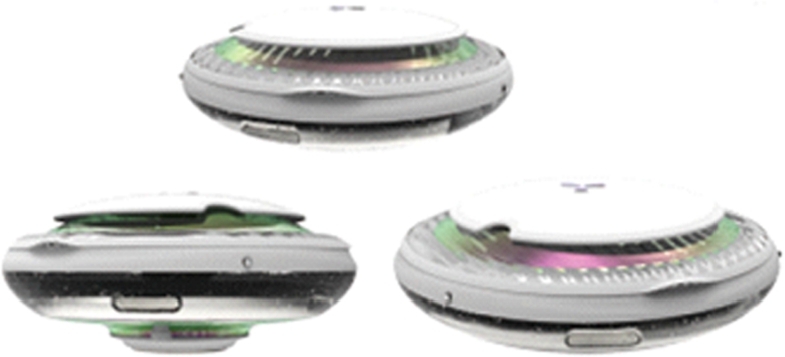
Renderings show.

**Figure 7:**
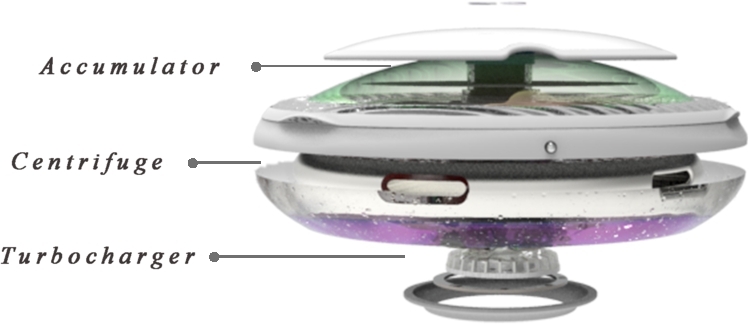
Explosion pattern.

**Figure 8:**
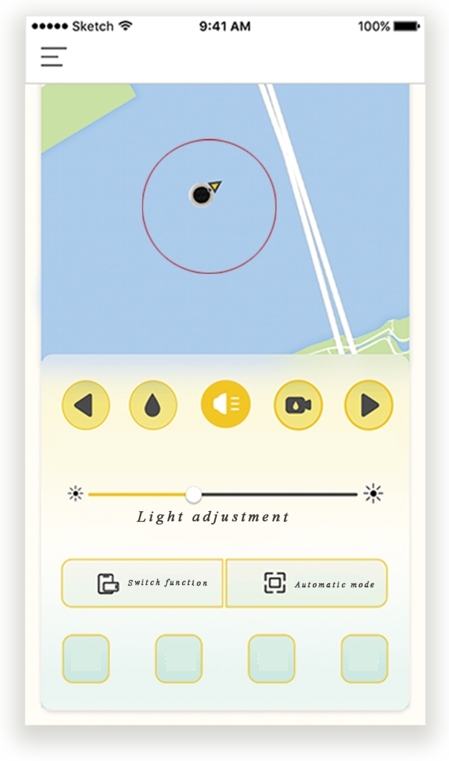
Remote control interface.

Next, the design description of this product is introduced. As shown in Figure 6, Figure 7, this is an offshore oil harvesting product design based on sustainability and developed using a combination of entropy weights and the VIKOR method. As shown in Fig. 8, the machine is capable of autonomous outdoor oil collection tasks based on intelligent algorithms, remote control technology, and physical oil and water separation in a specific operating environment. The appearance of the product is integrated, as the aerodynamic guiding principle of the streamline shape to balance the structure and function. Compared with the existing similar equipment of other types, the product designed in this paper has automatic path planning, automatic height adjustment, emergency hedging and other functions. At the same time, it can be combined with clean energy (solar energy) to operate, with a higher level of safety and intelligence.

### 4.4 Scheme comparative analysis

In order to validate the effectiveness and correctness of the entropy weight and VIKOR-based design solution under the sustainable framework, we made a comparison of the presented methodology with other existing traditional decision-making approaches, such as fuzzy comprehensive evaluation method, gray correlation analysis method, and TOPSIS method. The program evaluation and ranking results from these three methods are shown in Table 8.

**Table 8:** Comparison and analysis results of scheme evaluation values of various methods.

Method	Scheme 1	Scheme 2	Scheme 3	Sort
The proposed method	0.000	1.000	0.592	scheme 1 > scheme 3 > scheme 2
Fuzzy comprehensive evaluation method	0.340	0.328	0.332	scheme 1 > scheme 3 > scheme 2
TOPSIS method	0.025	0.291	0.137	scheme 1 > scheme 3 > scheme 2
Grey correlation analysis method	0.805	0.682	0.645	scheme 1 > scheme 2 > scheme 3

Which, as shown in Table 8, the fuzzy comprehensive assessment approach is expressed as a composite score, the higher the data score, the merrier the program. The TOSIS approach is denoted by the terms of positively ideally solved data, the fewer the data scores, the merrier the program. The gray correlation analysis approach is denoted by the terms of gray correlation score data, the higher the data score, the merrier the project. It is apparent from Table 8 that Option 1 is always the best choice. The ranking of the scenarios is consistent, except for scheme 2 and scheme 3, which have deviations in the gray correlation analysis method, and the results of this data prove the practicability and reasonableness of our recommended scheme as well as the method.

Compared with the other three programs, the approach presented in this article has the following three advantages:

1. Compared with the fuzzy integrated assessment methodology, the fuzzy comprehensive assessment method assigns the relative priority of per index component, there is a certain subjective uncertainty, and it can't solve the issue of duplication of assessment information resulting from the relevance among the index elements: the entropy value method adopted in the paper can reduce the subjective factors in the assignment process, to establish a more objective design program evaluation of the weighting model, so that the data results are more accurate. The difference between the programs of the fuzzy comprehensive evaluation method is small, and the data performance is stabilized.
2. Compared with the TOPSIS methodology, the appraisal value calculated by the TOPSIS approach are not very different, which is not conducive to the obvious discovery of the optimal program. While in the ranking sequence of the VIKOR approach, comparisons are usually made through cluster utility values, regret values and combined utility values, so as to complete the evaluation of the program's degree of superiority or inferiority ranking, the optimal program that can be obtained by the methodology is the nearest to the ideal value. As indicated in Table 8, there is little difference in the value of the programs calculated by the TOPSIS methodology, and when faced with a variety of program decisions, the method will reflect a greater advantage.
3. Comparison between the method in the paper and the gray correlation analysis method, gray correlation analysis approach generally involves inviting a number of experts in the field to evaluate the options and then derive the gray correlation numeric, which is more impartiality and the decision value of each option varies less. Table 8 points out that the difference between the maximum and minimum values calculated by the method is 0.16, while data with insignificant gaps are not facilitated more unbiased judgment on the part of policymakers, when there are more decision-making options. As shown in Table 8, the data obtained by the proposed method have a large difference, which is conducive to decision-makers to quickly sort the values and screen the scheme.

## 5 Conclusion

### 5.1 Management and theoretical implications

In addition, our findings provide insights for managers. When managers consider introducing new products in markets where buyers and end users are not necessarily the same, promoting the technology of the new product appears to be an effective marketing strategy. In addition, promoting a product's sustainable recycling of resources can mitigate the negative effects of perceived risk for new products. These efforts are noteworthy because our results suggest that people's attitudes toward a product directly influence behavioral intentions to use that product. Ultimately, in the context of situational awareness and environmental stewardship, the physical non-toxicity of the product and the recycling of resources could help the country to generate positive profits and a new industry chain.

The theoretical implications are as follows. Cognitive Processes: The 1st goal of this study is to elucidate the effect of sustainable elements of cognitive processes on the VIKOR and entropy weight methods. We found that using the VIKOR method we were able to rank the evaluation solutions according to their quality and obtain the ideal solution closest to the design goal, as well as using the entropy weight method to obtain a more objective weighting of the indicators. And sustainable factors can influence the direction of product positioning. Therefore, the influence of product subordinate frames and categories on the research method should be improved in product design, products in different specific situations require different design attribute frames, and developers should have good judgment and attribute knowledge of the field under study.

Design process: The 2nd goal of this study focuses on the impact of cognitive process elements on the conclusions of data modeling during the design process. We combine the entropy right and VIKOR approaches to conduct a comprehensive study on product case studies. During the calculations, we found a new correlation: in the ranking of the main influences on the product, Functional form, Energy saving and environmental protection, and technical level, are the main influencing points of the product performance. Of these, Energy saving and environmental protection are the main elements in the concept of sustainability. Therefore, we suggest that the product functions of the calculated model should be ranked to maximize the product performance during the calculation process.

Decision process: the 3rd goal of the present study focuses on the consistency test of the newly proposed method, verifying that the designed product can pass the test of traditional design research methods, and convincing the acceptance of the new method through the decision-making process. We used this synthetic method for model input and compared the results with other similar methods, and the experimental results verified the validity and reasonableness of the method, and through the analogy of the data results, Option 1 was always the most preferred choice. This decision-making process can provide data testing assurance for the method and guarantee the use of the designed product.

### 5.2 Limitations and future research topics

The study had several limitations. First, the generalization of our findings is limited to the subjectivity and uncertainty of the product design notion development period. It is not possible to fully consider other situations in the product time, such as the density of sea water in different sea areas and the distribution of ocean currents. Because different environmental relationships are different in nature, future studies may add additional variables relevant to that particular study context.

Second, experimental practice can more clearly demonstrate the causal relationship of the relationship between structures. For example, in the algorithm may be in the general method variance and bias. Therefore, the robustness of our findings can be enhanced by confirmation with other research methods.

Finally, the considerations for sea surface oil collection in this study are not comprehensive enough. For example, the evaluation part of the whole design process is completed by expert evaluation scoring, which increases the personal subjective factor of expert scoring and may lead to less comprehensive data. In addition, we used the determination of weight values by entropy weighting of the program to exclude the subjective preference of the decision maker, which to a certain extent ignored the auxiliary and corrective role of the decision maker for the design program. Therefore, we will further improve the method in the subsequent research. We will combine the comprehensive factors of the decision maker to evaluate the design scheme scientifically and reasonably, screen out the best design scheme, and further deepen the data optimization problem and related mechanics and mechanical principles.

It is worth noting that on the basis of the current study of marine oil collection methods, the future research direction can also go deeper into the problem of marine nuclear pollution, hoping to be able to solve more practical problems and bring more practical value and hope for the environment of human society.

## CRediT authorship contribution statement

**Suwen Luo:** Writing – review & editing, Writing – original draft, Software, Methodology, Investigation, Formal analysis. **Pengrui Yang:** Writing – review & editing, Resources.

## Declaration of Competing Interest

The authors declare that they have no known competing financial interests or personal relationships that could have appeared to influence the work reported in this paper.

## Data Availability

The data are not publicly available.
